# Co-infection of Cytomegalovirus and Epstein-Barr Virus in an Immunocompetent Patient: A Case Series and Literature Review

**DOI:** 10.7759/cureus.47599

**Published:** 2023-10-24

**Authors:** Osamah AlAmeen, Areej Mohammed, Mohanad Faisal, Samah Kohla, Ahmad Abdulhadi

**Affiliations:** 1 Internal Medicine, Hamad General Hospital, Doha, QAT; 2 Internal Medicine, Hamad Medical Corporation, Doha, QAT; 3 Laboratory Medicine and Pathology, Hematology Section, National Center for Cancer Care and Research (NCCCR) Hamad Medical Corporation, Doha, QAT

**Keywords:** ebv-associated hepatitis, hepatitis, viruses, cmv ebv, cytomegalovirus-cmv

## Abstract

Cytomegalovirus (CMV) infection or Epstein-Barr virus (EBV) infection in immunocompetent patients usually resolves without treatment. However, it can cause severe symptoms that can last for several weeks, especially in immunocompromised patients. Indications for antiviral immunocompetent individuals with CMV disease are not well-established. Here, we report two cases who had concomitant CMV-EBV infection. The first patient ultimately received anti-CMV therapy with significant improvement in symptoms and labs. The second patient had a milder disease course and was treated conservatively.

## Introduction

Infectious mononucleosis (IM) due to Epstein-Barr virus (EBV) or cytomegalovirus (CMV) mononucleosis are common infections throughout the world, with a 60%-100% seroprevalence worldwide. Primary infection in immunocompetent persons is normally asymptomatic or may result in mild and self-limiting infection. The self-limiting course of infection typically includes malaise, protracted fever, mild liver-function abnormalities, and lymphocytosis with atypical lymphocytes that occur in ~ 10% of immunocompetent adults [1]. Rarely, immunocompetent patients may develop a severe infection that manifests with multiple organ involvement, co-infection with other viruses could be the explanation for severe and multi-systemic infections [2]. The simultaneous infection by multiple herpesviruses is extremely rare, and because of this, there are limited existing cases published in the literature.

Herein, we report a case series of immunocompetent patients who presented with continuous spikes of fever and high liver enzymes, eventually after a thorough investigation turned serologically positive for the simultaneous presence of CMV and EBV infections.

Thus, this case series aims to highlight that co-infection via CMV and EBV or with other herpesviruses can be found in immunocompetent individuals and should be considered as a differential diagnosis, given that such events may not be exclusive of immunocompromised patients.

## Case presentation

Case 1

A 24-year-old with an unremarkable past medical history presented with a three-week history of fever, chills, and generalized weakness. His symptoms were associated with anorexia and dry cough as well. His fever did not respond to paracetamol or an empiric seven-day course of augmentin given at a primary health center. He also had a weight loss of 7 kg due to his concomitant loss of appetite. There were no sick contacts or history of travel or animal contact within this period. A review of other systems was unremarkable.

His vital signs were significant for persistent fevers reaching 38.5 C and tachycardia of 120 beats/min. A physical examination showed no lymphadenopathy or hepatosplenomegaly and was mainly unremarkable.

**Figure 1:**
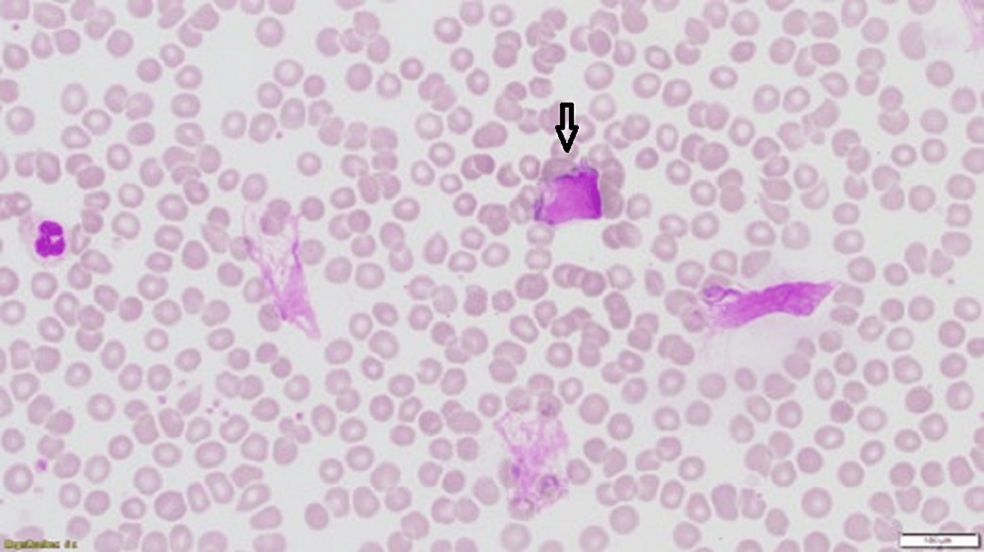
The peripheral blood smear shows normochromic normocytic red cells with lymphocytosis, many pleomorphic reactive atypical lymphocytes, and many smudge cells (Giemsa stain x40) in Case 1

**Figure 2:**
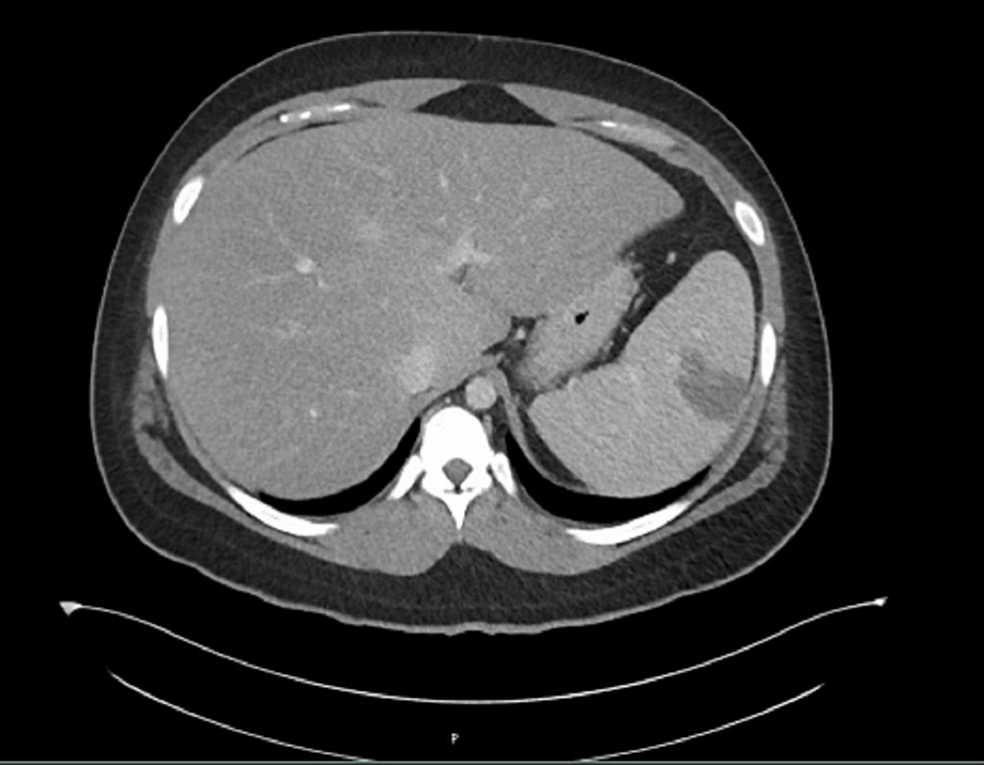
Splenomegaly with hypodensities and the appearance of a large hypodensity at the lateral subcapsular region in Case 1

He was empirically started on ceftriaxone and azithromycin, which were escalated 48 hours later to piperacillin-tazobactam due to persistent fever. Initial blood tests revealed leukocytosis with left shift and high liver enzymes (Table 1). Blood cultures and infection workup, including hepatitis, HIV serologies, malaria, TB, and brucella, were negative. However, the peripheral smear showed atypical lymphocytes (Figure 1) and further workup showed CMV and EBV viremia (Table 2). Abdominal CT showed splenomegaly with hypodensities (Figure 2). immunocompromised state was excluded as well, with negative HIV and autoimmune diseases. The patient was started on IV ganciclovir for five days, and antibiotics were discontinued. The patient showed clinical improvement and the fever subsided with liver enzyme improvement and his viral loads significantly decreased. After that, the patient was discharged on oral valganciclovir and was to be followed as an outpatient. He was symptom-free at the two-week follow-up and remained clinically stable.

**Table 1:** Laboratory tests for Case 1 WBC (white blood cells), Hb (hemoglobin), AST (alanine aminotransferase), AST (aspartate aminotransferase), INR (International normalized ratio)

Blood tests		Normal Values
WBC	15.4 x10^3/uL	4-10
Hb	13.1 gm/dL	13-17
AST	256 U/L	0-41
ALT	185 U/L	0-41
Alkaline phosphatase	237 U/L	40-130
INR	1.2	
Bilirubin total	42 umol/L	0-21
Bilirubin direct	37 umol/L	0-5

**Table 2:** EBV and CMV PCR levels on diagnosis and after five days of IV ganciclovir in Case 1 EBV (Epstein-Barr virus), CMV (Cytomegalovirus), PCR (polymerase chain reaction)

Blood tests		Normal Values
EBV PCR	2,535 IU/mL	Not detected
CMV PCR	74,528 IU/mL	Not detected
After 5 days of treatment		
EBV PCR	172 IU/mL	Not detected
CMV PCR	1,265 IU/mL	Not detected

Case 2

A 40-year-old female with an unremarkable past medical history presented with a five-day history of fever, flu-like symptoms, and abdominal discomfort. Her symptoms were associated with nausea and anorexia, which then progressed to jaundice. Her vital signs were normal, and her physical examination showed no lymphadenopathy or hepatosplenomegaly and was mainly unremarkable apart from jaundice.

Her lab tests were significant for high liver enzymes along with hepatosplenomegaly on abdominal imaging (Table 3). Further testing showed positive EBV and CMV immunoglobulin M (IgM) antibodies. However, subsequent polymerase chain reaction (PCR) testing showed positive EBV with high titer and negative CMV, and her HIV testing along with autoimmune screening were negative (Table 4). The patient was treated conservatively with intravenous fluids and anti-emetics and recovered without requiring anti-viral therapy. On follow-up, she remained asymptomatic and her liver function tests were normalized.

**Table 3:** High liver enzymes in case 2 WBC ( white blood cells), AST (alanine aminotransferase), AST (aspartate aminotransferase)

Blood tests		Normal Values
WBC	5.7 x10^3/uL	4-10
Total bilirubin	74 umol/L	0-21
AST	283 U/L	0-41
ALT	373 U/L	0-41
Alkaline phosphatase	395 U/L	40-130

**Table 4:** Showing positive IgM antibody for CMV and EBV but negative CMV PCR in Case 2 EBV (Epstein-Barr virus), CMV (Cytomegalovirus), IgM (immunoglobulin M), IgM (immunoglobulin M)

Blood tests		Normal Values
EBV Capsid antigen IgM	Reactive	Not reactive
CMV Ab IgM	Reactive	Not detected
EBV PCR	9,841 IU/mL	Not detected
CMV PCR	Not detected	Not detected

## Discussion

Cytomegalovirus causes a wide spectrum of diseases in healthy and immunosuppressed hosts. The majority of healthy individuals who develop CMV infection are able to recover within a short length of time, with no adverse sequelae. However, sometimes, symptoms can be prolonged with fever lasting for more than three weeks in immunocompetent patients with primary CMV infection [3]. Severe CMV infection in immunocompetent patients can carry a significant risk in this population and may be more common than previously assumed and it needs to be treated actively [4]

Simultaneous EBV-CMV infection is uncommon and is usually found in immunosuppressed patients. In a study of 190 pediatric patients with infectious mononucleosis who have multiple pathogens simultaneously, only seven patients had CMV-EBV infection with classic features of IM along with liver function abnormalities [5].

In another review, 28 hospitalized patients with mononucleosis syndromes tested positive for CMV and EBV IgM antibodies and around 50% of them had multiple co-morbidities and were immunocompromised [6]. Possible explanations by the authors for dual positivity included false positive antibodies of either of them, acute CMV-EBV infection, and sequential acute infections by EBV/CMV in either order with prolonged IgM positivity from the first infection, which is the most likely explanation in the second case described [7,8]. Our first patient could have developed EBV reactivation in the context of an acute CMV infection, and it could be similar to the case described by Olson where their patient was immunocompetent but recovered slowly without the use of antivirals [9,10].

Several prior reports highlight the significant mortality and morbidity associated with untreated CMV infection in immunocompetent patients with faster clinical improvement after anti-CMV therapy [11,12].

Our second patient was treated with IV ganciclovir for five days and then oral valganciclovir for three weeks, and his condition improved significantly after IV antiviral therapy. Oral valganiclovir alone can also be effective, as the main concern of ganciclovir is system toxicity [12,13]. Its availability has enabled the quick transition from intravenous to oral therapy in suitable CMV-infected patients to continue management in the outpatient setting.

## Conclusions

Co-infection of EBV and CMV in an immunocompetent patient is uncommon and can sometimes result in a diagnostic dilemma. Primary CMV viremia can occur in immunocompetent patients. Treatment with appropriate anti-virals should be considered to prevent complications and improve symptoms. However, further studies will be required to assess the need for anti-CMV therapy in immunocompetent patients with symptomatic CMV infections.
